# Galectin-1 Is Implicated in the Protein Kinase C ε/Vimentin-Controlled Trafficking of Integrin-β1 in Glioblastoma Cells

**DOI:** 10.1111/j.1750-3639.2008.00227.x

**Published:** 2010-01

**Authors:** Shannon Fortin, Marie Le Mercier, Isabelle Camby, Sabine Spiegl-Kreinecker, Walter Berger, Florence Lefranc, Robert Kiss

**Affiliations:** 1 Laboratory of Toxicology, Institute of Pharmacy, Univesité Libre de Bruxelles (ULB)Brussels; 2 Aurémie SPRLTubize, Belgium; 3 Department of Neurosurgery, Wagner Jauregg HospitalLinz, Austria; 4 Institute of Cancer Research, Department of Medicine I, Medical University of ViennaVienna, Austria; 5 Department of Neurosurgery, Erasme University HospitalBrussels, Belgium

**Keywords:** Galectin-1, Glioblastoma, Integrin beta1, Migration, Trafficking

## Abstract

Cell motility and resistance to apoptosis characterize glioblastoma (GBM) growth and malignancy. In our current work we report that galectin-1, a homodimeric adhesion molecule and carbohydrate-binding protein with affinity for β-galactosides, is linked with cell surface expression of integrin β1 and the process of integrin trafficking. Using immunofluorescence, depletion of galectin-1 through both stable knockdown and transient-targeted small interfering RNA (siRNA) treatment induces an intracellular accumulation of integrin-β1 coincident with a diminution of integrin-β1 at points of cellular adhesion at the cell membrane. Galectin-1 depletion does not alter the gene expression level of integrin-β1. Transient galectin-1 depletion effectuates as well the perinuclear accumulation of protein kinase C epsilon (PKCε) and the intermediate filament vimentin, both of which have been shown to mediate integrin recycling in motile cells. Our results argue for the involvement of galectin-1 in the PKCε/vimentin-controlled trafficking of integrin-β1. The understanding of molecular mediators such as galectin-1 and the pathways through which they drive the cell invasion so descriptive of GBM is anticipated to reveal potential therapeutic targets that promote glioma malignancy.

## INTRODUCTION

Gliomas are the most common primary brain tumors, among which glioblastoma multiforme (GBM) is the most malignant form characterized by its heterogeneity and aggressive invasive behavior into normal brain tissue; GBM is classified as World Health Organization grade IV, and to date there is no cure (25, 26). Galectin-1 is a multifaceted promoter of glioma malignancy (8); galectin-1 instigates increased glioma invasiveness through modifications to the actin cytoskeleton and increased Ras homolog gene family, member A small guanosine-5′-triphosphate (GTP)ase expression (6) and facilitates adhesion to the extracellular matrix (ECM) by cross-linking glycoproteins (integrins) to ECM components (6). Galectin-1 expression levels in glioma correlate directly with tumor grade across astrocytic tumors and inversely with patient survival in high-grade astrocytic tumors (6). Furthermore, galectin-1 expression is higher in the invasive areas of glioblastoma xenografts (5, 32). We have previously shown that addition of exogenous galectin-1 to the culture media of U87 glioblastoma cells stimulates cell motility, and that inhibition of galectin-1 via microinjection of antisense galectin-1 significantly decreases cell motility (6). Stable knockdown of galectin-1 in U87 glioblastoma cells alters their gene expression pattern by increasing, among other genes, the expression of genes involved in adhesion including integrin alpha 7 and integrin alpha 9 (7).

Integrins are known to play a significant role in the progression of cancer cell malignancy through their involvement in cell adhesion, motility and intracellular signaling (1, 14, 16), with emphasis on the role of the beta 1 integrin subunit in gliomas (2, 11, 29). There is now much evidence to support that directed cell migration requires the recycling of adhesion receptors through membrane trafficking pathways (9, 31). Integrins are heterodimeric membrane spanning receptors; they consist of an α and a β subunit, each of which traverses the membrane once with most of each polypeptide in the extracellular space (16). Integrins are trafficked first through an internalization step and then, after sorting at the early endosomes, are sent through an exocytosis pathway (9, 31). Integrin internalization proceeds either through clathrin-mediated vesicle formation or through caveolar endocytosis at the site of cholesterol-rich membrane domains (9, 31), and is thought to be directed per signaling motifs on the integrin cytodomain (9, 30). After sorting, integrins are shuttled back to the cell surface via either the Rab4-controlled short-loop pathway, or via the Rab11 long-loop pathway that proceeds through the perinuclear recycling complex (9, 31). Several studies have linked integrin trafficking to cell motility; many regulators of integrin trafficking (including adenosine 5′-triphosphate-ribosylation factor 6, pyruvate dehydrogenase kinase isozyme 1 and dynamin) are found in high levels in invadopodia (9), and increased rates of internalization and endocytosis are found at the leading edge of migrating cells (9, 19).

Many protein kinases, as well as microtubules, actin and intermediate filaments act to facilitate integrin trafficking (9, 19, 30, 31). Among these, protein kinase C epsilon (PKCε) has been shown to regulate the recycling of endocytosed integrin-β1 for return to the plasma membrane in motile fibroblast cells (17). PKCε-dependent phosphorylation on several N-terminal serine residues of the intermediate filament vimentin allows for the plasma membrane return of integrin-β1 (18). PKCε is an isoform of PKC and has been shown to translocate to the cell membrane at points of matrix adhesion in HeLa cells (10) and to contribute to haptotaxis (17).

In this study we describe the galectin-1 regulation of integrin-β1, PKCε and vimentin localization in human glioma cells. A decrease in galectin-1 expression induces an analogous intracellular accumulation of these collaborators in receptor recycling. Here we elucidate, in part, the PKCε/vimentin-dependent pathway that defines galectin-1-mediated glioma cell migration via integrin-β1.

## MATERIALS AND METHODS

### Cell cultures and compounds

The Hs683 [American Type Culture Collection (ATCC) code HTB-138], U87 (ATCC code HTB-14), U373 (ATCC code HTB-17) and T98G (ATCC code CRL-1690) human GBM cell lines were obtained from the ATCC (Manassas, VA, USA) and maintained in our laboratory as detailed previously (5–7, 32). We also made use of four primocultures established in our facilities as detailed previously (3). Briefly, tumor specimens were transferred during surgery into culture medium [RPMI-1640 with 20% fetal calf serum (FCS), 1% glutamine, 1% penicillin/streptomycin; Gibco, Invitrogen, Merelbeke, Belgium]. The tissue was minced and washed with physiologic NaCl solution to remove red blood cells. After passage 5, cells were grown in a culture medium containing 10% FCS without antibiotics. These primocultures have been labeled GL-5, GL-16, GL-17 and GL-19 in the current study. They are part of a collection of several hundreds of GBM primocultures established at the Department of Neurosurgery of the Wagner Jauregg Hospital (Linz, Austria).

### Inhibition of galectin-1 expression in human glioblastoma cells

#### Transient transfection of anti-Gal1 small interfering RNA (siRNA)

The sense sequence of the anti-Gal1 siRNA (Eurogentec, Seraing, Belgium) used in the current work was 5′-gcugccagauggauacgaadtdt-3′ and the antisense sequence was 5′-uucguauccaucugg cagcdtdt-3′(21, 22). A corresponding scrambled siRNA (scr) was used as a control (sense: 5′-cuacgaugcugcuuagcucdtdt-3′ and antisense: 5′-gagcuaagc agcaucguagdtdt-3′) (21, 22). The siRNA and scr control we made use of in the current study have been proven to be the most efficient among several siRNAs and scr controls tested in a previous study (21). The antisense and sense strands of the siRNA were annealed by the manufacturer in 50 mM Tris, pH 7.5–8.0, 100 mM NaCl in diethylenepyrocarbonate (DEPC)-treated water. The final concentration of siRNA duplex was 100 µM. The antisense and sense strands of the scrambled control were annealed in the same way. Hs683 and U87 cells were either left untreated or were transfected with calcium phosphate (Kit ProFection® mammalian transfection system, Promega, Leiden, the Netherlands) over 16 h with either anti-galectin-1 siRNA or scrambled siRNA (day 0). On day 2, each group of cells was pooled and replated either into new flasks to decrease cell confluency for subsequent time points, or onto glass coverslips to be further analyzed by immunofluorescence (IF) as detailed previously (21, 22). On days 5, 7 and 9, Hs683 and U87 cells were scraped into cold phosphate-buffered saline (PBS) buffer for RNA extraction, lysed directly in boiling lysis buffer (10 mM Tris pH 7.4, 1 mM Na_3_O_4_V, 1% sodium dodecyl sulfate, pH 7.4) for protein extraction, or fixed with 4% formalin for IF analysis. Galectin-1 siRNA shutdown efficiency was confirmed by both Western blot (WB) and IF analyses (using an anti-galectin-1 antibody purchased from Preprotech TebuBio, Boechout, Belgium).

#### Stable transfection of antisense galectin-1 vectors

In addition to, but separate from the transient transfection with anti-Galectin-1 siRNA, U87 cells were also stably transfected with antisense galectin-1 vectors to shutdown galectin-1 expression. These U87 cells that were stably transfected and used in the current work are issued from a previous study in which the transfection protocol and controls used are fully detailed (7).

#### Protein expression measurements

WB and IF analyses were performed as detailed previously (2, 21, 22). For IF, cells were either permeabilized with Triton X-100 0.3% for 20 minutes in PBS + bovine serum albumin 0.1%, or left non-permeabilized and control experiments included the omission of the incubation step with the primary antibodies (negative control). For WB, the integrity and equal loading of the extracts was assessed using a tubulin antibody. For WB and IF the primary antibodies used were as follows: anti-galectin-1 (WB dilution 1/500, IF dilution 1/100; Preprotech TebuBio), anti-integrin α9β1 (dilution 1/50; Chemicon, Biognost BVBA, Heule, Belgium), anti-vimentin (WB dilution 1/1000, IF dilution 1/100; Chemicon, Biognost BVBA), anti-PKCε (WB dilution 1/500, IF dilution 1/50; BD Transduction Laboratories, Erembodegem, Belgium), and anti-tubulin (dilution 1/2000; Abcam, Cambridge, UK). The staining for fibrillar actin was performed using fluorescent phallacidin-conjugated with Alexa Fluor 488, whereas Fluor 594-conjugated DNaseI (Molecular Probes, Invitrogen, Merelbeke, Belgium) was used to stain globular actin, as detailed elsewhere (6). Secondary antibodies were purchased from Pierce (PerbioScience, Erembodegem, Belgium) for the WBs and from Molecular Probes (Invitrogen) for fluorescent detection (Alexa fluor-conjugated antibodies). WBs were developed using the Pierce Supersignal Chemiluminescence system (PerbioScience).

### Genomic analysis

Hs683 cells were transfected twice with scrambled (control siRNA) or siRNA directed against galectin-1 according to the procedure detailed earlier. RNA extraction and the determination of the quality and the integrity of the extracted RNA were assessed as detailed elsewhere (21, 22). Full genome analysis was performed on day 5 post-transfection at the VIB MicroArray Facility (UZ Gasthuisberg, Catholic University of Leuven, Leuven, Belgium) using the Affymetrix Human Genome U133 set Plus 2.0 (Affymetrix, High Wycombe, UK). The microarray data analysis was carried out as detailed elsewhere (21, 22). Indeed, we used the previous set of data from the studies detailed in Le Mercier *et al*(21, 22).

### Standard reverse transcription-polymerase chain reaction (RT-PCR)

The procedure used was identical to that described previously (2, 24). RT-PCR was performed using the following primers: integrin α9 forward 5′-tccctaaacatctctatctcc-3′ and reverse 5′-agtacgactttcttctttagca-3′; integrin β1 forward 5′-gactgttctttggatactagtact-3′ and reverse 5′-cagctacaattggaatgatgtc-3′; and actin forward 5′-ctaagtcatagtccgcctag-3′ and reverse 5′-aaatcgtgcgtgacattaagg-3′. The PCR conditions for Integrin α9 were as follows: predenaturation, 4 minutes, 94°C; PCR amplification, 35 cycles at 94°C, 30 s (denaturation), 59°C, 30 s (annealing), 72°C, 1 minute (extension); and final extension: one cycle at 72°C, 10 minutes. The PCR conditions for integrin β1 were as follows: predenaturation, 4 minutes, 94°C; PCR amplification, 35 cycles at 94°C, 30 s (denaturation), 58°C, 30 s (annealing), 72°C, 1 minute (extension); and final extension: one cycle at 72°C, 10 minutes. For actin, the PCR conditions were as follows: predenaturation, 4 minutes, 94°C; PCR amplification, 25 cycles at 94°C, 30 s (denaturation), 62°C, 30 s (annealing), 72°C, 1 minute (extension); and final extension: one cycle at 72°C, 10 minutes. PCR products were resolved on a 1% agarose gel, and visualized using Sybr Safe reagent (Invitrogen, Eugene, OR, USA).

### Quantitative RT-PCR (qRT-PCR)

The determination of integrin α9 and integrin β1 mRNA expression in three human glioma cell lines by qRT-PCR was performed against a standard curve established through serial dilutions (10^8^ to 10^2^ copies/µL) of the PCR products generated with specific primers. qRT-PCR was performed using the following primers: integrin α9 forward 5′-tccctaaacatctctatctcc-3′ and reverse 5′-tgattccggtgatggac-3′, and integrin β1 forward 5′-gactgttctttggatactagtact-3′ and reverse 5′-cagctacaattggaatgatgtc-3′ The quantitative PCR reactions were carried out with 20 ng of purified complimentary deoxyribonucleic acid in the LightCycler thermocycler (Roche Diagnostics, Vilvoorde, Belgium) using LC-Faststart DNA Master SYBR Green 1 (Roche Diagnostics). After amplification, data analysis was carried out using the “fit points” algorithm of the LightCycler quantification software (Roche Diagnostics).

## RESULTS

### Galectin-1 siRNA is most effective at silencing galectin-1 gene expression between day 5 to day 9 post-transfection

**Figure 1:**
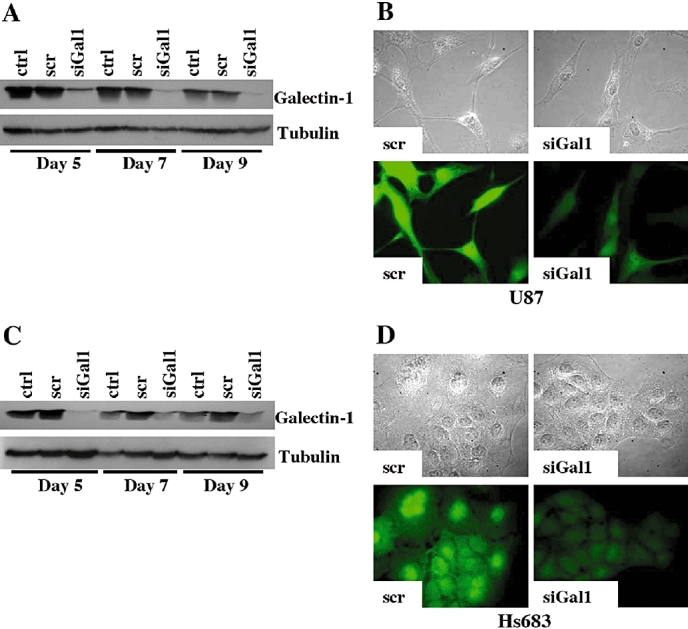
*Transient transfection of galectin-1 using targeted small interfering RNA (siRNA) decreases intracellular galectin-1 protein.* WB analysis of galectin-1 expression levels at days 5, 7 and 9 post-transfection in control (ctrl), scramble-transfected (scr), and galectin-1-transfected (siGal1) U87 (**A**) and Hs683 (**C**) cells. Immunofluorescence (IF) analysis of galectin-1 expression levels in U87 day 7 post-transfection (**B**) and Hs683 day 5 post-transfection (**D**) using scrambled or anti-galectin-1 siRNA. Top rows are the corresponding bright fields to the IF images.

Galectin-1-targeted siRNA was transiently transfected into both U87 and Hs683 glioblastoma cells. Levels of intracellular galectin-1 protein were measured by both WB and IF; galectin-1 was effectively diminished from day 5 to day 9 post-transfection in U87 cells (Figure 1A,B) and in Hs683 cells (Figure 1C,D) in comparison with control and scramble-transfected cells.

### Integrin-β1 is more widely expressed across glioma cell lines than integrin-α9, and neither the expression of integrin-β1 nor of integrin-α9 was modified following transient transfection of siRNA targeting galectin-1

**Figure 2:**
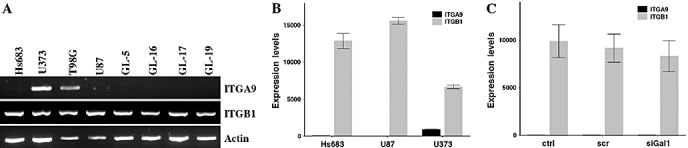
*Integrin-α9 versus integrin-β1 expression*. **A.** Integrin-α9, integrin-β1 and actin expression across eight glioma cell lines by reverse transcription-polymerase chain reaction (RT-PCR) analysis. **B.** Quantitative RT-PCR analysis of integrin-α9 versus integrin-β1 expression in glioma cell lines Hs683, U87 and U373. **C.** Hs683 expression levels of integrin-α9 and integrin-β1 under control (ctrl), scramble-transfected (scr) or galectin-1 siRNA-transfected (siGal1) conditions obtained through microarray analysis using the Affymetrix Human Genome U133 set Plus 2.0.

As galectin-1 has been shown to modulate glioma cell adhesion and migration (5, 6, 32), we sought to define the relationship between galectin-1 and the integrin most emphasized in GBM cell migration, integrin-β1 (2, 11, 29). We obtained an antibody recognizing integrin-α9/β1 and confirmed that this antibody was more specific to integrin-β1. We studied expression of each integrin across eight glioma cell lines and found that whereas integrin-β1 was universally expressed (Figure 2A), integrin-α9 was expressed only in the U373 and T98G cell lines (Figure 2A) by RT-PCR. This result was further confirmed by use of quantitative RT-PCR in the Hs683, U87 and U373 glioma cell lines; integrin-β1 is expressed among all three cell lines, whereas integrin-α9 is only expressed in U373 and at lower levels than integrin-β1 (Figure 2B). For the remainder of the studies we have chosen to continue with the cell lines Hs683 and U87 to be sure that our antibody is integrin-β1 specific. We then used microarray analysis with the Affymetrix Human Genome U133 set Plus 2.0 to determine whether transient transfection of siRNA targeting galectin-1 affected either integrin-α9 or integrin-β1 gene expression. Among Hs683 control, scramble-transfected or anti-galectin-1-transfected cells, we saw no significant change in the gene expression of either integrin gene (Figure 2C) indicating that galectin-1 expression levels do not regulate the expression of integrin-β1 or integrin-α9.

In a previous study (7) we observed that long-term (several months) knockdown of galectin-1 in U87 glioma cells increased expression of alpha 9 integrin, whereas in the present study we did not observe effects on integrin expression levels (including alpha 9) following short-term (several days) knockdown of galectin-1. It thus seems that prolonged downregulation of galectin-1 in glioma cells could be compensated by the overexpression of certain integrins, including integrin-α9.

### Stable and transient knockdown of galectin-1 expression leads to a loss of beta 1 integrin in focal adhesion complexes and to an increase in integrin β1 intracellular levels

**Figure 3:**
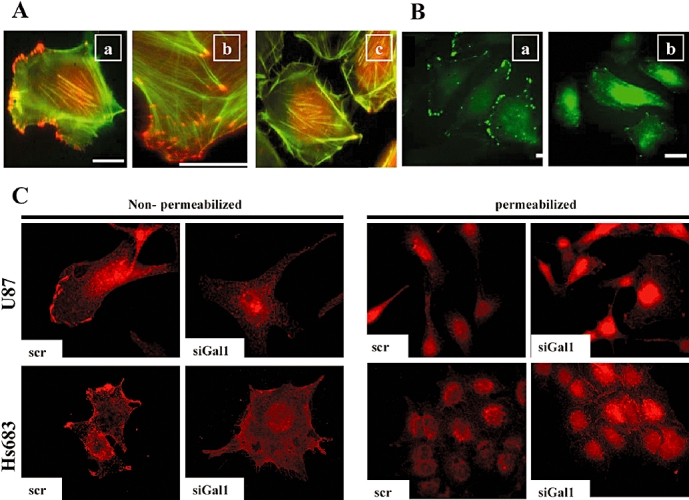
*Galectin-1-targeted siRNA induces both a decrease of integrin-β1 at the edges of the cell membrane and an increase in the intracellular accumulation of integrin-β1*. **A.** U87 control wild-type cells (**Aa,Ab**) and U87 cells containing stably transfected antisense galectin-1 vectors (**Ac**) were co-stained under non-permeabilized conditions for integrin-β1 [the red staining at the tip of actin stress fibers (in green fluorescence)], fibrillar actin (green) and globular actin (red staining inside the cells). **Ab.** Higher magnification of the control wild-type (**Aa**) image. **B.** U87 control wild-type cells (**Ba**) and U87 cells containing stably transfected antisense galectin-1 vectors (**Bb**) were stained under permeabilized conditions for integrin-β1 only (in green), without any staining to reveal actin (**Ba,Bb**). **C.** U87 and Hs683 cells were transiently transfected with either scrambled siRNA (scr) or siRNA targeted against galectin-1 (siGal1). Cells were stained for integrin-β1 (in red) under both permeablized and non-permeabilized conditions on either day 5 post-transfection (Hs683) or day 7 post-transfection (U87).

As galectin-1 is involved in adhesion complexes (8), we asked whether decreasing galectin-1 expression altered the pattern of integrin expression at the leading edge of cell migration. In both control U87 and U87 cells stably expressing an antisense galectin-1 vector we left the cells non-permeablized and co-stained them with integrin-β1 and fibrillar (polymerized) and globular (non-polymerized) actin. In control cells, we saw integrin-β1 localization at points of focal adhesion complexes along the cell membrane at the edge of fibrillar actin staining (Figure 3Aa,Ab), whereas in stable galectin-1 knockdown cells the cell membrane localization of integrin-β1 was lost (Figure 3Ac). In Figure 3Aa–Ac, the red fluorescence inside the cells corresponds to globular actin (DNaseI staining), not to integrin-β1. Thus, in comparison, we stained permeablized control and stable galectin-1 knockdown cells with integrin-β1, and found that the galectin-1 knockdown cells contained an intracellular accumulation of integrin-β1, with the cell membrane staining once again lost in comparison with control cells (Figure 3Ba,Bb).

We further explored this intracellular integrin-β1 accumulation phenomenon in both U87 and Hs683 cells transiently transfected with scrambled or galectin-1-targeted siRNA. Similar to the stable knockdown results, in both U87 and Hs683 cells non-permeabilized integrin-β1 was found stained at sites of focal adhesion complexes along the cell membrane of scramble-transfected cells, a pattern that was lost in galectin-1 siRNA-transfected cells (Figure 3C, non-permeabilized). Coincident again with results from galectin-1-stable knockdown, when U87 and Hs683 cells were permeabilized and stained for integrin-β1, an increased intracellular staining pattern was observed (Figure 3C, permeabilized). These data show that decreasing galectin-1 expression leads to the localization change in integrin-β1 from sites at the cell membrane to an intracellular accumulation.

### Summarizations of integrin trafficking and protein export pathways to determine potential points of galectin-1 regulation of integrin-β1 localization

**Figure 4:**
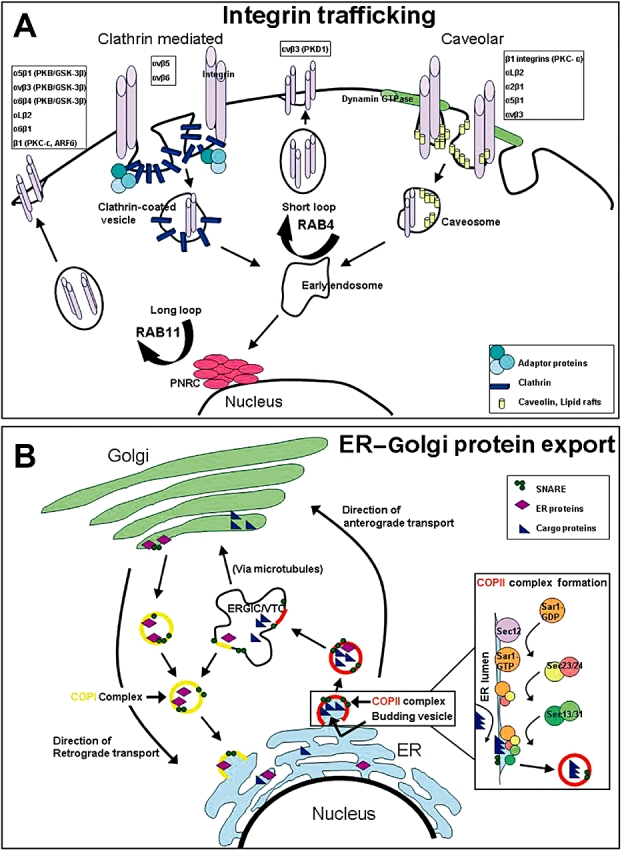
*Integrin trafficking and endoplasmic reticulum (ER) to Golgi protein export pathways.***A.** Integrin trafficking depicting the two main pathways of internalization, clathrin-mediated and caveolar, as well as the pathways for recycling. Clathrin-mediated internalization utilizes clathrin coated pits, whereas caveolar internalization utilizes dynamin GTPase to form vesicles containing lipid rafts and caveolin protein. Recycling occurs through fusion to early endosomes and proceeds either via Rab4 signaling through a short loop pathway or via Rab11 signaling through a long loop pathway where proteins pass through the perinuclear recyling complex. Respective integrins and their trafficking mediators are listed above the suggested pathways that they utilize (9, 17, 19, 30, 31). **B.** ER to Golgi protein export depicting coat protein complex II (COPII)-mediated anterograde transport and coat protein complex I (COPI)-mediated retrograde transport between the ER and the Golgi complex. COPII assembly commences with Sar1 G-protein activation by the Sec12 guanine nucleotide exchange factor, followed by the recruitment of the Sec23–24 heterodimer that recognizes ER export signals on cargo proteins for inclusion in vesicles. The Sar1-Sec23–24 complex recruits the Sec13–31 heterodimer that facilitates final budding and vesicle formation from the ER. Vesicles proceed to the ER–Golgi intermediate compartment (ERGIC), which is also called vesicular tubular clusters (VTC), the complex of which transports proteins to the Golgi destination for eventual final export (15, 20, 23). GTP = guanosine-5′-triphosphate; SNARE = N-ethylmaleimide-sensitive fusion protein attachment protein receptor. PKC = protein kinase C.

As treatment with galectin-1 siRNA causes a loss of membrane localized and an increase in intracellular localized integrin-β1 (Figure 3), we sought to determine if galectin-1 played a key role in directing either protein export pathways pertaining to integrins, or in integrin trafficking. We mapped out the processes of both integrin trafficking (Figure 4A) and of traditional protein export (Figure 4B), and compiled a list of the major genes directing these systems. Using the microarray analysis with the Affymetrix Human Genome U133 set Plus 2.0 on Hs683 cells left untreated, scramble-transfected, or transfected with siRNA targeting galectin-1, we inquired whether decreasing galectin-1 altered the gene expression of any of the genes on our list. We found a small set of genes whose expression was altered by at least 1.5-fold in either the control versus galectin-1 siRNA ratio or in the scramble siRNA versus galectin-1 siRNA ratio (Table 1). These data were merely to suggest whether integrin trafficking and/or protein export pathways may facilitate galectin-1-induced changes in integrin-β1 cellular localization, but did not solidify any particular genes as front-runners for potential mediators in these processes. These data show that galectin-1-targeted siRNA alters only to a small degree the gene expression of genes involved in integrin trafficking and protein export.

**Table 1:** Genes involved in integrin trafficking and export whose expression is modified following galectin-1 small interfering RNA (siRNA) treatment.

Gene Name	Symbol	Ct: galectin-1 siRNA-transfected (siGal1)	Scrambled siRNA: siGal1
Coatomer protein complex, subunit alpha	COPA	1, 7	1, 5
Coatomer protein complex, subunit gamma	COPG	1, 5	1, 5
V-akt murine thymoma viral oncogene homolog 3 (protein kinase B, gamma)	AKT3	1, 6	1, 7
V-akt murine thymoma viral oncogene homolog 3 (protein kinase B, gamma)	AKT3	1, 1	1, 5
Protein kinase C, epsilon	PKCε	1, 4	1, 9
SEC24-related gene family, member D (*S. cerevisiae*)	SEC24D	3, 8	3, 1
SEC24-related gene family, member D (*S. cerevisiae*)	SEC24D	3, 9	3, 0
SEC24-related gene family, member A (*S. cerevisiae*)	SEC24A	1, 5	1, 5
SEC24-related gene family, member A (*S. cerevisiae*)	SEC24A	1, 4	1, 5
SEC31-like 1 (*S. cerevisiae*)	SEC31L1	2, 2	1, 6
SEC31-like 1 (*S. cerevisiae*)	SEC31L1	1, 6	1, 6
SEC31-like 1 (*S. cerevisiae*)	SEC31L1	1, 6	1, 6
RAB5A, member RAS oncogene family	RAB5A	1, 5	1, 4

### Galectin-1-targeted siRNA treatment leads to perinuclear accumulation of both PKCε and the intermediate filament vimentin

**Figure 5:**
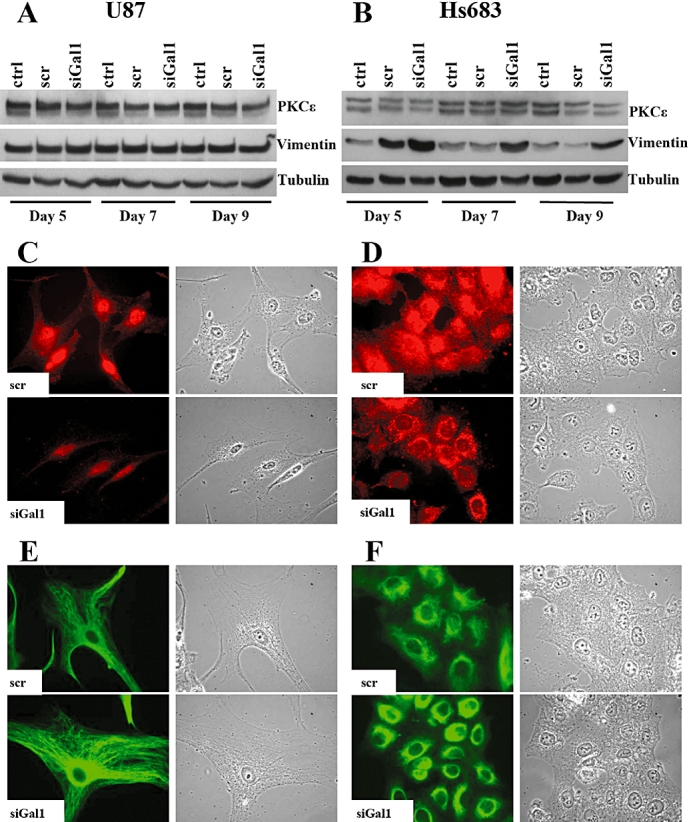
*Western blot (WB) and immunofluorescence (IF) analysis of vimentin and protein kinase C epsilon (PKCε) protein expression levels; galectin-1-targeted siRNA increases only vimentin protein levels in Hs683, induces increased perinulear amassment of both PKCε and vimentin, and diminishes the diffuse cytoplasmic staining of PKCε*. WB analysis of PKCε and vimentin protein expression levels in U87 (**A**) or Hs683 (**B**) cells untreated (ctrl), scramble-transfected (scr) or galectin-1 siRNA-transfected (siGal1) at days 5, 7 and 9 post-transfection. IF analysis of PKCε localization in U87 (**C**) or Hs683 (**D**) scr or siGal1 at either day 5 (Hs683) or day 7 (U87) post-transfection. IF analysis of vimentin localization in U87 (**E**) or Hs683 (**F**) cells scr or galectin-1 siRNA-transfected (siGal1) at either day 5 (Hs683) or day 7 (U87) post-transfection. In all IF figures right columns represent the corresponding bright field images.

To begin to assess biochemically whether any of the genes whose expression was modified upon treatment of siRNA targeting galectin-1 were involved in integrin-β1 cellular localization changes, we inquired if whether, similar to integrin-β1, there were changes in the localization of the trafficking and export-related genes. Using the genes affected from the gene expression microarray study under galectin-1-targeted siRNA treatment as the first potential candidates (Table 1), we first selected the gene PKCε to study for its known role in integrin-β1 trafficking (17). We assessed through WB analysis the expression of PKCε in both U87 and Hs683 cells under conditions of siRNA targeting galectin-1 at days 5, 7 and 9 post-transfection, and found no significant changes in PKCε protein expression levels (Figure 5A,B). We then performed IF studies of PKCε localization in both U87 and Hs683 under conditions of siRNA targeting galectin-1 at day 5 (Hs683) or day 7 (U87) post-transfection. Scramble-transfected cells showed a diffuse staining pattern for PKCε in the cytoplasm, whereas the localization under galectin-1 siRNA conditions shifted towards only a strong perinuclear staining in both U87 (Figure 5C) and Hs683 (Figure 5D) cells.

As PKCε-controlled integrin traffic has been shown to require the intermediate filament vimentin (18), we further explored the protein expression and localization patterns of vimentin. WB analysis of vimentin levels in U87 cells transfected with galectin-1 siRNA at days 5, 7 and 9 post-transfection revealed no change under siRNA conditions (Figure 5A), however, vimentin levels in similarly transfected Hs683 cells appeared to increase upon treatment with siRNA-targeting galectin-1 (Figure 5B). We then performed IF studies of vimentin localization in both U87 and Hs683 under conditions of siRNA targeting galectin-1 at day 5 (Hs683) or day 7 (U87) post-transfection. We observed a similar staining phenomenon as seen with PKCε; in cells treated with siRNA-targeting galectin-1, there was an increased accumulation of perinuclear vimentin as compared with scramble-transfected U87 (Figure 5E) and Hs683 (Figure 5F) cells. These data show that decreasing galectin-1 focuses the localization of both PKCε and vimentin to the perinuclear cell region.

## DISCUSSION

Our current study reports the galectin-1 regulation of integrin-β1, PKCε and vimentin localization. We observed in both galectin-1 stable and galectin-1 transient knockdown cells a loss of integrin-β1 on the cell membrane at points of focal adhesion complexes coincident with an increase in intracellular levels of integrin-β1. Integrin-β1 has been shown to play a role in glioma malignancy; using antisense integrin-β1 mRNA, Paulus *et al* showed that integrin-β1 is required for the diffuse invasion of glioma cells (29). As galectin-1-targeted siRNA did not significantly change the expression of integrin-β1 in comparison with control and scramble-transfected cells (Figure 2), the loss of membrane integrin-β1 and intracellular accumulation of integrin-β1 observed in IF under galectin-1-targeted siRNA is indeed caused by shifts in localization, and not merely an increase in expression of the integrin. Galectin-1 may therefore direct, in part, malignant progression of glioma by regulating the role of integrin-β1 through changes in localization. Furthermore, galectin-1 has been shown in numerous studies to bind integrin-β1, modulating adhesion and FAK activation (13, 27, 28), and inhibiting cellular growth through the Ras–MEK–ERK pathway (12). Galectin-1 may therefore use a multifaceted approach in influencing integrin-β1 activity; intracellular galectin-1 may work to promote integrin-β1 recycling, and extra-cellular galectin-1 may work to promote integrin-β1-mediated cell attachment and motility.

In this study we also reported the effects of galectin-1 depletion on PKCε and vimentin localization. In both cell lines tested, transient transfection of siRNA-targeting galectin-1 actuated increased perinuclear localization of both PKCε and vimentin. In galectin-1 siRNA-transfected cells, PKCε also lost the diffuse cytoplasmic staining pattern seen in scramble-transfected cells. Although, as stated previously (17) that PKCε has been shown to direct integrin-β1 trafficking, it has furthermore been shown to direct the integrin-dependent adhesion and motility of human glioma cells, inducing focal adhesion and lamellipodia formation with the suggestion that the RACK1 scaffolding protein mediates integrin beta chain interaction with activated PKCε(4). This association provides the suggestion that galectin-1 may mediate glioma invasion, in part, through PKCε-directed integrin-β1 adhesion and motility.

Ivaska *et al* provided a scheme for PKCε and vimentin involvement in integrin recycling whereby vimentin oligomers associate with internalized integrin compartments allowing for both the recruitment of PKCε and the subsequent phosphorylation of vimentin by PKCε, the process of which leads to the release of the vimentin-PKCε complex and the return of integrins to the plasma membrane (18). As we have shown a perinuclear aggregation of both PKCε and vimentin under galectin-1 depletion that is similar to the vesicular aggregation of these two molecules seen by Ivaska *et al* under inhibition of PKCε activity that, in their study, lead to decreased integrin recycling, we can implicate galectin-1 in mediating the PKCε–vimentin-controlled integrin-β1 recycling pathway in glioma (18). In addition, overexpression of PKCε has been shown in primary pediatric anaplastic astrocytoma (grade III) tumor samples and derived cell lines, and high levels of PKCε have been seen in both GBM (grade IV) and gliosarcoma tumor samples, but not in pilocytic astrocytomas (grade I) (33). Similarly, galectin-1 expression levels are significantly higher in the diffusely infiltrating astrocytic tumors than in pilocytic astrocytomas (6). The high expression of both PKCε and galectin-1 in high-grade glioma, and their suggested collaboration towards the malignant phenotype, marks both molecules as exciting targets for therapeutic intervention.

Although galectin-1 does appear to affect the process of integrin-β1 recycling by influencing the localization of PKCε and vimentin, this may not be the only mechanism that actuates the intracellular accumulation of integrin-β1 under decreased galectin-1 conditions. The depletion of galectin-1 gene expression levels through siRNA also revealed gene candidates involved in the process of protein export; indeed the gene expression of Sec24, a member of the coat protein complex II that mediates vesicle formation in endoplasmic reticulum exit sites and transport to vesicular-tubular clusters in the endoplasmic reticulum (ER)-Golgi intermediate compartment (ERGIC) (Figure 4B) (15, 20, 23), is downregulated under depleted galectin-1 conditions (Table 1). Although Sec24 has not been shown to have specificity for mediating integrin export, depletion of Sec24 isoforms—with Sec24A having the most significant impact—led to disruptions in ER export through decreased ability to recognize ER export signals on the ERGIC-53 cargo protein (34). It is possible that galectin-1 also works to influence Sec24 expression and therefore play a role in ER export pathways. It would be interesting to study the effect of galectin-1 depletion on the ability of Sec24 incorporation into the coat protein complex on vesicles forming from the ER, and on the ability of Sec24 to read ER export signals in order to direct cargo protein transfer to the ERGIC.

This study strengthens the role of galectin-1 in facilitating the malignant invasive phenotype of glioblastoma. Galectin-1 has been implicated in glioma adhesion and migration (5–7, 32), and in this report we suggest that the galectin-1 influence on integrin-β1, PKCε, and vimentin localization provides evidence that galectin-1 utilizes integrin trafficking pathways to impel glioma malignancy.
